# Gut Microbiota in HIV Infection: Implication for Disease Progression and Management

**DOI:** 10.1155/2014/803185

**Published:** 2014-06-12

**Authors:** Felix Chinweije Nwosu, Ekaterina Avershina, Robert Wilson, Knut Rudi

**Affiliations:** ^1^Hedmark University College, 2306 Hamar, Norway; ^2^Department of Chemistry, Biotechnology and Food Sciences, Norwegian University of Life Sciences, P.O. Box 5003, 1432 Aas, Norway

## Abstract

Survival rates among HIV patients have significantly improved since the introduction of antiretroviral therapy (ART) in HIV management. However, persistent disease progression and clinical complications in virally suppressed individuals point to additional contributing factors other than HIV replication; microbial translocation is one such factor. The role of underlying commensal microbes and microbial products that traverse the intestinal lumen into systemic circulation in the absence of overt bacteraemia is under current investigation. This review focuses on current knowledge of the complex microbial communities and microbial markers involved in the disruption of mucosal immune T-cells in the promotion of inflammatory processes in HIV infections. Unanswered questions and aims for future studies are addressed. We provide perspective for discussing potential future therapeutic strategies focused on modulating the gut microbiota to abate HIV disease progression.

## 1. Introduction

The human immunodeficiency virus (HIV), a lentivirus and causal agent of acquired immunodeficiency syndrome (AIDS), was first described in the early 1980s [1]. HIV causes extensive, multifactorial dysregulation of the immune system in infected individuals. The immune system becomes compromised through a progressive depletion of CD4+ T-lymphocytes (T-helper cells), macrophages, and dendritic cells of the immune system, which gives rise to opportunistic infections and disease. The rapid destruction of these CD4+ T-cells primarily occurs in the mucosal tissue such as the gut associated lymphoid tissues (GALT), harboring an abundance of activated memory CD4+ T-cells [2]. This destruction progresses to the peripheral tissues and blood and continues within the next 7 to 10 years [3, 4].

The proposed involvement of gut microbiota in inflammatory processes that lead to disease progression such as inflammatory bowel disease (IBD) [5] has been widely reported in the literature [6]. There are over 100 trillion bacterial cells in the human gut [7] and they serve as providers of important metabolites for the host. Moreover, they are active participants involved in the modulation of host gene expression, maintenance of epithelial integrity, and mediation of inflammatory and antimicrobial responses [8, 9]. Hence, the cumulative insult on the human innate and adaptive immune system by HIV infection has been linked to dysbiosis in the human gut bacteria [10, 11]. As such, the translocation of intestinal bacteria and products into systemic circulation has implicated roles in inflammation and invariably immune activation [10, 12].

Several investigations point to the resident gut microbiota as an integral driver of pathologic inflammation that persists in patients even during the administration of antiretroviral therapy (ART) [9, 13]. However, the mechanisms of human gut microbiota involvement in inflammatory processes that may influence disease progression in HIV are still poorly elucidated. This understanding will be important in the further development of effective therapies.

Recent studies implicate a potential causal relationship between the composition of gut microbes and various human disease conditions [14]. Observed clinical manifestations are attributed to the immune system, which, through complex interactions, shapes and structures gut microbial communities [7]. In addition, nutritional or metabolic factors also influence these structures [10]. Though capable of exerting beneficial effects, the microbiota can also exacerbate disease progression by directly or indirectly enhancing viral infection. The microbiota also plays vital roles in the maturation of the specific immune system of the lymphoid organs, which also serve as the first line of defence of the gastrointestinal mucosa [14].

This review provides an update and a discussion on the influence of the human gut microbiota in HIV infection and disease progression. In the same vein, the influence of prebiotics and probiotics in the management of HIV infections will be examined. Finally, unanswered questions relating gut microbes and HIV progression and future perspectives for achieving effective therapeutic strategies will be highlighted.

## 2. Gut Microbiota in Early HIV Infections

By definition, early HIV infection is the stage from infection (undetectable viral load) and through the acute phase (clinical manifestations of HIV observable), 3–5 days after infection. At this stage, rapid viral replication, intense immune response, immune destruction, viral diversification [15, 16], and CD4 cell count of greater than 200 cells/*μ*L [17–20] are observed. The stage of infection is critical in the investigation of microbiotal composition, and atypical microbiotas have been observed in early stages of HIV infection [21].

In HIV infections, mucosal integrity is pertinent to disease progression, and similarly, viral infections are initiated at points exposed to the surrounding environment, rife with resident microorganisms [22]. Therefore, HIV infection would have to negate both gut bacteria and epithelial barriers to propagate infection. The GALT, which harbors over 60 % of a person's CD4+ T-cells, is impaired during the early stages of HIV infection, suggesting an important role of the associated mucosal microbiota in HIV infection and progression [12, 21]. In one study, higher levels of Enterobacteriales and* Bacteroidales* correlated to the levels of GALT CD4+ T-cell depletion and immune activation observed in HIV patients compared to healthy controls [10]. Conversely, Gori et al. [21] also observed overrepresentation of pathogenic species,* Candida albicans* and* Pseudomonas aeruginosa* in early HIV patients. These species have been implicated in opportunistic infections observed in HIV-infected subjects [10, 21]. Species such as bifidobacteria and lactobacilli, with propensities to boost gut immune function by modulating the innate immune system in pathogenic infection, are depleted in the faecal microbiota of untreated HIV patients [20, 21, 23].

A shift in proportions of pathogenic and commensal microbes relative to viral load has been observed, in some studies involving other early stage HIV patients, either treated or untreated [24, 25]. Similarly, a recent study elucidated imbalances in adherent gut bacterial communities in HIV-infected subjects, either naive or treated with highly active antiretroviral therapy (HAART) [25]. According to reports by Vujkovic-Cvijin et al. there is a significant enrichment and depletion of the Enterobacteriaceae family and Bacteroidia class, respectively, in untreated HIV positive subjects compared to treated subjects which either exhibited a profile of bacterial composition similar to HIV^−^ (healthy controls) or HIV^+^ (infected) [25]. Thus, the progression of inflammation symptoms in HIV patients showing suppressed viral loads during ART could be explained by this effect of gut bacteria species deemed to be proinflammatory. This observation further underlines the potential involvement of microbiotal composition in HIV progression.

Thus, studies elucidating imbalances in the gut microbiome associated with HIV infection have invariably incited an interest to investigate the mechanisms of microbiota involvement in the progression of this disease. Hence, current and future research aims to explain the observed shift in gut composition from protective species to proinflammatory, disease-inducing bacterial species that create an environment capable of promoting viral replication and chronic immune activation. The specific clustering of microbiotas in diseased and healthy control subjects and the dissimilar compositions observed in patients treated with ART may also suggest an important role for bacteria affecting viremia following HIV infection [25]. However, the manner in which the microbiota elicits immune activation in HIV subjects is still poorly understood, and experimental studies seeking to link gut resident bacteria with this phenomenon are highly anticipated in the future.

## 3. Gut Microbiota in HIV Progression

Due to increased permeability of the intestinal barrier observed in HIV-infected subjects, translocating microbes or microbial products have been partially attributed to HIV disease progression. Recent findings support this view and progress towards understanding the role microbial translocation plays in HIV/AIDS pathogenesis was achieved by determining levels of systemic lipopolysaccharides (LPS) in circulation [12]. LPS is a known activation factor of monocytes modulating innate and adaptive immunity. Although it does not directly account for all microbial translocation [19], Brenchley et al. [12] demonstrated that it is bioactive* in vivo* in mice. However, contradictory results have been published that link microbial translocation and HIV disease and progression in human subjects or animal models [26]. Redd et al. [27] demonstrated no correlation between LPS levels and counts of soluble CD14 (sCD14), a coreceptor in the recognition of bacterial LPS in systemic circulation. In another study, increased levels of LPS were associated with HIV infection in late stages [28, 29]. These contrary findings indicate that microbial translocation is not the sole driver of HIV progression. On the other hand, incongruence among these studies may also result from differences in experimental approaches in determining the levels of LPS and sCD14 [26]. Either way, the massive loss of GIT lamina propria CD4+ T-cells, the first target of HIV, may imply an important role of the gut microbiota in HIV pathogenesis and progression in later stages of the disease [30]. Similarly, an earlier study reported the existence of a polymicrobic flora in blood samples of HIV-infected patients showing a poor response to HAART and highlighting a damaged intestinal barrier as the driving cause of microbial translocation [9, 23]. In another study, persistent T-cell hyperactivation was attributed to disease progression in known immunologic nonresponders to HAART and invariably hampered CD4+ T-cell reconstruction [31]. Hence, in late stages, the persistence of peripheral T-cell immune activation and CD4 decline, coinciding with the richness of microbes or microbial products in systemic circulation, has been implicated as a predominate determinant of the disease progression rate in HIV-infected patients [32]. Also, Hunt et al. showed that T-cell activation remains persistent even during HAART therapy and can be exacerbated with coinfections such as hepatitis C [33]. This highlights that effective treatment of HIV comorbidities is essential for management of HIV disease progression. Although levels of bacterial rDNA in plasma correlate with LPS levels and magnitude of immune activation [34], the mechanisms by which HIV elicits T-cell immune activation are still largely unknown [35].

As in other infections [36] microbial translocation occurs in HIV infection [37] and certainly contributes to immune activation [12]. Supposedly, other underlying factors other than HIV are also implied in HIV progression, which may include microbial translocation, directly or indirectly, in HIV progression [9]. Although ART also seems to reduce the levels of microbial translocation, restoring some GIT barrier function, it does not completely revert HIV infection [12]. However, further investigation is needed to firmly establish microbial translocation as a cause of HIV progression. It is currently proposed that microbial translocation is a direct consequence of HIV replication and its chronic immune activation of the hosts' immune system irrespective of viral load reconstruction [12] and can otherwise be predictive of disease severity [29]. It will therefore be of interest to investigate any direct or indirect links of gut microbes and microbial products in other non-AIDS related disorders such as liver, cardiovascular, central nervous system, and cancer diseases among others. A consequential effect of microbial translocation is supported in most studies to date. For example, in a recent study, Manner et al. [18] observed a correlation between LPS levels and hypertension in HIV subjects independent of CD4 cell counts. These non-AIDS diseases have been significant contributors to morbidity and mortality among HIV infected patients with greater microbial translocation reported due to damaged intestinal epithelium [17, 38].

Although a direct relationship between microbial translocation and HIV disease progression may seem to exist [26, 39] especially in the chronic phase of the infection [12], it remains clear that HIV itself fuels disease progression by initiating a proinflammatory environment in the GIT and inflicting damage on the mucosal barrier.

## 4. Disease Management by Pro- and Prebiotics

The compromised gut barrier in HIV infections results in the loss of the mutually beneficial coevolving association of gut epithelium and adherent bacteria. Therefore, intervention therapy targeting the gut microbiota to curb HIV progression has been proposed. Modulating the composition of the microbiota is gaining prominence as a strategy for future therapies. This is based on findings associating the resident bacteria with immune activation. As such, studies have been ascribed to certain bacterial species, roles in maintaining epithelial mucosal integrity [40], and the early maturation of the adaptive immune system [7, 21], and these are being exploited in progut formulations [41]. HIV subjects are known to have a compromised gut barrier function and the observation that* Bifidobacterium* and* Lactobacillus* species are depleted among the gut bacteria populations in such patients [23] is consistent with earlier studies attributing improved gut barrier and immune function to these species [42]. Essentially, certain strains of these beneficial microbes have proven effective in maintaining gut barrier function to ameliorate various diseases associated with GIT inflammation [1]. Adequately administered probiotics (live microorganisms that exert effects on health) can express antimicrobial compounds such as bacteriocins that may kill or inhibit the growth of pathogenic microbial species in the gut, thus, conferring a net health benefit [1, 41]. Prebiotics are also used to modulate resident gut homeostasis [41, 42]. Prebiotics are nondigestible food components such as fiber or oligosaccharides, which selectively promote growth of beneficial bacterial species. In this way, pathogenic gut bacteria that maybe implicated in opportunistic infections, a hallmark of HIV infection, are eliminated or reduced. Treatments termed synbiotics, involving a combination of both probiotics and prebiotics, have also been reported [41]. Although the mechanisms by which these treatments achieve mucosal reconstruction are not completely elucidated, it is believed to occur through the activity and recruitment of natural killer cells [43]. Also, increases in tight junction proteins as a response to the administration of VSL#3 probiotics (combination of eight bacterial species belonging to* Lactobacillus*,* Streptococcus,* and* Bifidobacterium* genera) have been demonstrated* in vivo *and* in vitro* [44].

At present, few studies report the use of prebiotics, probiotics, or synbiotic dietary supplements in HIV therapy, but their future use as a novel treatment strategy is gaining support. According to a preliminary, placebocontrolled clinical trial by Gori et al. [42], a mix of three oligosaccharide prebiotics were effective in modulating gut microbiota reconstruction through the stimulation of bifidobacteria growth and consequent reduction of pathogenic species. It resulted in decreased sCD14 and LPS levels and activation of CD4+ T-cell and NK cells for at least 12 weeks in HIV-infected adults naïve to ART. In a similar study involving a probiotic yogurt, the invasion of gastrointestinal mucosa by pathogenic bacteria was completely inhibited [45]. The* L. rhamnosus* GR-1 stain supplement used in this study exhibited a significant mucosal adhesive potential, as well as reduction of ART drug-induced stomach pain in patients. This strain is generally recognized as safe (GRAS) by the American Food and Drug Administration, and it can, due to its adhesive properties, inhibit or promote microbial translocation. Therefore, the use of existing and novel probiotics formulations should be further investigated. Although long-term effects have not been firmly established, positive correlations in modulating gut bacteria and mucosa reconstruction were demonstrated in a 25-week study using capsules of* L. rhamnosus* GR-1 and* L. reuteri* RC-14 [43]. This study also showed that the time during which the treatment is administered may determine its measured efficacy.

An analysis of the key differentially expressed genes in the colon, in response to a probiotic/prebiotic cocktail mix in simian immunodeficiency virus (SIV) infected subjects, revealed a significant upregulation of APC-associated gene expression involved in T-cell immunity [46]. These macaques were subsequently treated with antiretroviral (ARV) drugs. This study therefore shows that, in addition to ARV, the synbiotic supplement has the potential to modulate GI immune function by increasing gut CD4+ T cells and reducing inflammation. However, not all bacterial or oligosaccharide mixes may be equally effective to clinically treat HIV infection. For example, a placebocontrolled, clinical study administering the synbiotic formulation Synbiotic 2000 that combines four probiotic strains (*Pediococcus pentosaceus* 5-33:3,* Leuconostoc mesenteroides* 32-77:1,* Lactobacillus paracasei* subsp.* paracasei* 19, and* Lactobacillus planetarium *2362) and four prebiotics of nondigestible, fermentable fibers (beta glucan, inulin, pectin, and resistant starch) for 4 weeks, to ART-treated HIV patients resulted in unaltered plasma bacteria DNA concentration and persistent immune activation, but levels of the gut probiotic* Lactobacillus* species improved [47].

Other kinds of gut modulating formulations are also on the rise. These orally ingested bioactive substances/mixes can be taken with food [48] or as medical food [49]. To examine the impact of orally delivered serum-derived bovine immunoglobulin (SBI) on HIV enteropathy, Asmuth et al. [49] recruited eight HIV patients on ART with chronic gastrointestinal symptoms. As stated, SBI was used distinctively in clinical dietary management as commercially available supplements, and it showed efficacy in modulating bowel movements and increasing the density of CD4+ lymphocytes in all subjects. These findings thus suggest that the modulation of the mucosa can have desirable effects in the restoration and promotion of duodenal function and intestinal repair [49].

## 5. Knowledge Gaps

Viral suppression by ART alone has been insufficient in the clinical management of HIV. Therefore, it is imperative to determine the role of the gut microbiota in HIV-induced pathology, as microbial translocation, immune activation, and gut inflammation may influence therapeutic efficacy. Similarly, since LPS levels are difficult to measure [19], methods presently employed to measure microbiota markers associated with HIV infection and/or progression such as the relationship between inflammation and translocation need to be explored irrespective of LPS levels.

Various studies concur that the microbiota is involved in HIV disease progression as a consequence of chronic immunodeficiency [26]. In this light, the incorporation of strategies targeting the microbiota in addition to the viral load may prove quite effective in future clinical HIV therapies. Probiotics, prebiotics or synbiotics have shown promising effects, though issues related to prolonged use, formulation effectiveness, and universal immune preservation in a geographically independent manner remain unresolved [41]. Although these formulations are deemed effective and improve gut function in HIV subjects, their role in modulating mucosal immunity, circumventing the Th17 epithelial barrier cells, is still largely unknown [22]. Also, clinical trials incorporating larger study cohorts will afford increased statistical bases to conclusively assess the effects of microbial supplements on clinical outcomes of HIV infection. Probiotics and/or prebiotics are inexpensive and would represent cost effective treatments for HIV patients, a clear advantage in a global perspective given the economic status of regions where HIV is most prevalent [41]. Similarly, novel therapeutic strategies would be of particular interest in noncommunicable HIV comorbidities (cardiovascular, bone, liver, central nervous system). These disorders have been highlighted as a major cause of morbidity even in ART-treated, HIV-infected patients. Given that the mechanisms underlying this comorbidity are proposed to be associated with immune activation and microbial translocation, correlations with effective therapeutic regimes should be pursued [50].

Other strategies could include directly treating microbes or microbial products in patients with LPS nonproducing bacteria or agents that bind LPS. Such would be aimed at negating monocyte activation and the consequent triggering of immune inflammation thereby curtailing further disease progression [1]. Effective agents directing intestinal tract immune activity such as the Toll-like cell receptors that respond to microbial stimuli may also be targeted for intact gut maintenance [12, 29]. However, existing knowledge of the mechanism and interaction between the gut microbiota and the mucosa are still lacking and would be fully explored. Better understanding of the complex microbiome in HIV infection will be crucial before the realization of these strategies for the future. This can be achieved by incorporating studies to expose immune tolerance strategies of patients with undetectable viraemia and the cases of HIV nonprogressors [51].

Future studies should include metagenomic evaluation to elucidate microbial genes implicated in HIV disease progression. Also, it would be beneficial to design and perform large descriptional studies of complex microbial communities in HIV patients. Microbes play crucial roles in human biology and harbor and express a greater number of genes in the body than the host itself. Deciphering their contribution will have a great impact on human health [11]. Recently,* Pseudomonas fluorescens* strain A506, a gut pathobiont marker, was isolated and shown to be able to transform tryptophan to kynurenine, thus overlapping the function of a human gene [4]. Furthermore, advances in metagenomic approaches can broaden our knowledge of microbial markers associated with HIV by taking advantage of the enumerated human gut gene catalogue, a part of the MetaHIT project [11]. In doing this, a direct implication of gut microbiota in HIV progression may be elucidated by characterising human and microbial gene functions and their overlaps.

## 6. Conclusions

In conclusion, HIV infections characteristically cause damage to the gut mucosa, weakening the epithelial barrier, compromising gut immunity, and exposing its host to a broad range of microbial bioproducts that have been implicated in the progression of HIV. Similarly, the gut microbiota is severely impacted and the altered bacterial communities play vital roles, directly or indirectly in HIV progression. ART alone does not effectively control microbial translocation through the gastrointestinal tract. The contribution of gut microbes in HIV disease progression has been explored but conclusive knowledge is lacking. Therefore, an increased understanding of correlations between the proinflammatory bacterial community composition and structure in the human gut and HIV infection status can contribute to an improved and holistic management of HIV progression.
